# Surveillance of multiple congenital anomalies; searching for new associations

**DOI:** 10.1038/s41431-023-01502-w

**Published:** 2023-12-05

**Authors:** Joan K. Morris, Jorieke E. H. Bergman, Ingeborg Barisic, Diana Wellesley, David Tucker, Elizabeth Limb, Marie-Claude Addor, Clara Cavero-Carbonell, Carlos Matias Dias, Elisabeth S. Draper, Luis Javier Echevarría-González-de-Garibay, Miriam Gatt, Kari Klungsøyr, Nathalie Lelong, Karen Luyt, Anna Materna-Kiryluk, Vera Nelen, Amanda Neville, Isabelle Perthus, Anna Pierini, Hanitra Randrianaivo-Ranjatoelina, Judith Rankin, Anke Rissmann, Florence Rouget, Geraldine Sayers, Wladimir Wertelecki, Agnieszka Kinsner-Ovaskainen, Ester Garne

**Affiliations:** 1 Population Health Research Institute, St George’s, University of London, London, UK; 2 Department of Genetics, University Medical Center Groningen, University of Groningen, Groningen, The Netherlands; 3 Children’s Hospital Zagreb, Centre of Excellence for Reproductive and Regenerative Medicine, Medical School University of Zagreb, Zagreb, Croatia; 4 Clinical Genetics, University of Southampton and Wessex Clinical Genetics Service, Princess Anne Hospital, Southampton, UK; 5 Congenital Anomaly Register & Information Service for Wales (CARIS) Public Health Knowledge and Research, Public Health Wales, Swansea, Wales UK; 6 Department of Woman-Mother-Child, University Medical Center CHUV, Lausanne, Switzerland; 7 Rare Diseases Research Unit, Foundation for the Promotion of Health and Biomedical Research in the Valencian Region, Valencia, Spain; 8 Epidemiology Department, National Institute of Health Doutor Ricardo Jorge, Lisboa, Portugal; 9 Department of Population Health Sciences, Georg Davies Centre, University of Leicester, Leicester, UK; 10 Department of Health of the Basque Government, Vitoria-Gasteiz, Spain; 11 Malta Congenital Anomalies Registry, Directorate for Health Information and Research, Guardamangia, Malta; 12 Department of Global Public Health and Primary Care, University of Bergen, Bergen, Norway; 13 Division of Mental and Physical Health, Norwegian Institute of Public Health, Bergen, Norway; 14 Université Paris Cité, CRESS, Équipe de recherche en épidémiologie obstétricale périnatale et pédiatrique (EPOPé), INSERM, INRA, Paris, France; 15 South West Congenital Anomaly Register, Bristol Medical School, University of Bristol, Bristol, UK; 16 Polish Registry of Congenital Malformations, Chair and Department of Medical Genetics, University of Medical Sciences, 61-701 Poznan, Poland; 17 Provincial Institute for Hygiene, Antwerp, Belgium; 18 Center for Clinical and Epidemiological Research, University of Ferrara, Ferrara, Italy; 19 Auvergne Registry of Congenital Anomalies (CEMC-Auvergne), Department of Clinical Genetics, Centre de Référence des Maladies Rares, University Hospital of Clermont-Ferrand, Clermont-Ferrand, France; 20 Unit of Epidemiology of Rare diseases and Congenital anomalies, Institute of Clinical Physiology, National Research Council, Pisa, Italy; 21 Service de Génétique Médicale et d’oncogénétique, Registre des Malformations Congénitales, Saint Pierre, La Réunion France; 22 Population Health Sciences Institute, Faculty of Medical Sciences, Newcastle University, Newcastle upon Tyne, UK; 23 Malformation Monitoring Centre Saxony-Anhalt, Medical Faculty Otto-von-Guericke University-Magdeburg, Magdeburg, Germany; 24 Brittany Registry of Congenital Anomalies, CHU Rennes, Univ Rennes, Inserm, EHESP, Irset (Institut de recherche en santé, environnement et travail) – UMR_S 1085, F-35000 Rennes, France; 25 Health Intelligence, Research and Development Health Service Executive, Dublin, Ireland; 26 OMNI-Net Ukraine Programs, Rivne, Ukraine; 27 European Commission, Joint Research Centre, Ispra, Italy; 28 Department of Paediatrics and Adolescent Medicine, Lillebaelt Hospital, University Hospital of Southern Denmark, Kolding, Denmark

**Keywords:** Epidemiology, Genetics research

## Abstract

Many human teratogens are associated with a spectrum of congenital anomalies rather than a single defect, and therefore the identification of congenital anomalies occurring together more frequently than expected may improve the detection of teratogens. Thirty-two EUROCAT congenital anomaly registries covering 6,599,765 births provided 123,566 cases with one or more major congenital anomalies (excluding chromosomal and genetic syndromes) for the birth years 2008–2016. The EUROCAT multiple congenital anomaly algorithm identified 8804 cases with two or more major congenital anomalies in different organ systems, that were not recognized as part of a syndrome or sequence. For each pair of anomalies, the odds of a case having both anomalies relative to having only one anomaly was calculated and the *p* value was estimated using a two-sided Fisher’s exact test. The Benjamini–Hochberg procedure adjusted *p* values to control the false discovery rate and pairs of anomalies with adjusted *p* values < 0.05 were identified. A total of 1386 combinations of two anomalies were analyzed. Out of the 31 statistically significant positive associations identified, 20 were found to be known associations or sequences already described in the literature and 11 were considered “potential new associations” by the EUROCAT Coding and Classification Committee. After a review of the literature and a detailed examination of the individual cases with the anomaly pairs, six pairs remained classified as new associations. In summary, systematically searching for congenital anomalies occurring together more frequently than expected using the EUROCAT database is worthwhile and has identified six new associations that merit further investigation.

## Introduction

Identifying new teratogens is one of the main goals of a congenital anomaly registry. As many known human teratogens are associated with a spectrum of congenital anomalies rather than an isolated anomaly [1–5], identification of cases with multiple congenital anomalies that occur together more frequently than would be expected due to chance alone is likely to be more sensitive in the detection of teratogens. Around three out of four fetuses with a congenital anomaly have an isolated anomaly [6]. Among the remaining fetuses many of these anomalies are due to a chromosomal anomaly (for example, Down syndrome), a single gene defect (for example, Noonan syndrome) or a known teratogen (for example, cytomegalovirus infection), but some fetuses have more than one major anomaly without known etiology. Multiple congenital anomalies may also occur as a consequence of a single primary anomaly (for example, the Potter sequence resulting from renal agenesis and with secondary lung hypoplasia and clubfoot). It is therefore important to identify cases with two or more congenital anomalies in different organ systems, where the pattern of anomalies has not been recognized as part of a syndrome, known association or sequence as these could indicate unknown teratogens or new associations. Around 2% of all births have a congenital anomaly, but multiple congenital anomalies occur in around 16 per 10,000 births, with specific combinations of anomalies being even rarer [7]. Therefore, it is necessary to analyze the data from large datasets with sufficient cases.

The aim of this study was to analyze data from the EUROCAT network of congenital anomaly registries collected from births between 2008 and 2016. A method to automatically identify all pairs or triplets of anomalies occurring more frequently than would be expected due to chance alone was developed. Once such pairs/triplets were identified, the literature was searched to determine if such pairs/triplets of anomalies had already been identified as being part of an association or sequence. Any new pairs of anomalies were examined in greater detail by the registries to determine if any genetic test results were available.

## Subjects and methods

The first step in the identification of multiple anomalies is correct case classification. The EUROCAT multiple congenital anomaly algorithm has been developed in collaboration between the EUROCAT Central Registry and the Coding and Classification Committee and continuously improved since 2004 [6]. The members of the Coding and Classification Committee are geneticists and pediatricians. The algorithm classifies cases into different groups based on ICD-10/British Paediatric Association (BPA) codes. The aim of the algorithm is to classify congenital anomaly cases into:

- Chromosomal syndromes: all cases where an unbalanced chromosomal anomaly has been diagnosed, irrespective of types of anatomically defined anomalies.
- Genetic and environmental syndromes: all cases are due to a single gene defect or a known environmental teratogen, irrespective of types of anatomically defined anomalies. This includes skeletal dysplasias and hereditary skin disorders.
- Isolated anomalies: all cases with one congenital anomaly/anomalies occurring in only one organ subgroup or with a known sequence where multiple congenital anomalies cascade as a consequence of a single primary anomaly.
- Multiple congenital anomalies: cases with two or more major congenital anomalies in different organ systems, where the pattern of anomalies has not previously been recognized as part of a syndrome or sequence.

Papers published in 2011 and 2014 describe the methodology and results of the first 2 years of data [6, 7]. The computer algorithm allocates 90% of all EUROCAT cases into classification groups (a), (b) or (c). Approximately 10% of cases are classified by the computer as potential multiple cases and these cases were reviewed by three EUROCAT geneticists to reach an agreement for classification as true multiple congenital anomaly cases (d) or allocation to another group. A web-based system for review of cases has been developed, which allows easy and fast review of many cases and transfer of the final decision back to the central database. If two geneticists agreed on a case classification, this was considered the final decision. If all three geneticists disagreed or one of them classified the case for query, the moderator made the final decision.

Thirty-two full-member registries covering 6,599,765 births provided 154,154 cases with one or more major congenital anomalies born between 2008 and 2016. Cases with chromosomal and genetic syndromes, skeletal dysplasias or hereditary skin disorders were excluded resulting in 123,566 cases for inclusion in this analysis.

### Statistical methods

Sixty EUROCAT congenital anomaly subgroups were used in the analysis (Appendix Table A); 57 specific congenital anomaly subgroups and three more general congenital anomaly subgroups (neural tube defects (NTDs), congenital heart defects (CHD) and Severe CHD [8]).

### Analysis of multiple congenital anomaly cases only

All cases classified above as multiple congenital anomaly cases were analyzed as follows. For each pair of anomalies (say A and B) the odds of a case having anomaly B given that it had anomaly A relative to the odds of a case having anomaly B given that it did not have anomaly A was calculated and the associated *p* value estimated using a two-sided Fisher’s exact test. (Note that the odds ratio for anomaly A given anomaly B is identical to the odds ratio for anomaly B given anomaly A—so only one test was performed for each anomaly pair). The relative odds were not calculated for pairs of anomalies included in the same organ or system (for instance ventricular septal defect (VSD) and any other cardiac anomaly). They were also not calculated for clubfoot with spina bifida or renal dysplasia as clubfoot is considered to arise as a result of the occurrence of spina bifida or renal dysplasia. Finally, they were not calculated for situs inversus and any cardiac anomaly as this association is part of the heterotaxy spectrum.

Multiple testing procedures were carried out using the Benjamini–Hochberg procedure to control the false discovery rate. This gave a corrected overall *p* value to determine statistical significance and thus adjusted *p* values were calculated. Pairs of anomalies with adjusted *p* values < 0.05 were examined further. The analysis was repeated for males and females separately as hypospadias is only present in males (33 cases of indeterminate sex and 489 cases with missing sex were excluded).

Logistic regression models were used to examine associations between three anomalies. Each anomaly in turn was regressed on two other anomalies and the interaction term provided an estimate of the odds ratio for all three anomalies given any of the other two anomalies. As before, sets of anomalies known to be related were excluded, and the Benjamini–Hochberg procedure to control the false discovery rate was applied to obtain adjusted *p* values.

### Analysis of all cases with an anomaly

The above analysis was repeated on the population of all anomaly cases (*n* = 123,566), not just those with multiple anomalies. The number of cases with both anomalies remains the same, but the number of cases with each individual anomaly increases due to the inclusion of cases with only one anomaly. The estimated relative odds were therefore inflated, and the *p* values reduced. We therefore only examined pairs of anomalies with adjusted *p* values < 0.01, rather than the <0.05 cut-off used above.

### Results of the statistical analysis

The EUROCAT multiple congenital anomaly algorithm followed by a review by three EUROCAT geneticists identified 8804 (7.1%; 95% confidence interval (CI): 7.0–7.3) cases for the 9 years with two or more anomalies out of all 123,566 cases without a genetic disorder. The proportion of multiple anomaly cases was greater in males (7.1%; 95% CI: 6.9–7.3) than in females (6.8%; 95% CI 6.5–7.0), though not statistically significant.

A total of 31 statistically significant positive associations between two EUROCAT subgroups were found when analyzing the cases with multiple congenital anomalies only and judging significance from an adjusted *p* value < 0.05. These results are very similar to those obtained by analyzing all cases with a congenital anomaly (rather than just those with multiple anomalies) and selecting those associations with an adjusted *p* value of <0.01 (Tables 1 and 2). The results were also similar when males and females were analyzed separately.

**Table 1:** Identification of 20 known associations amongst the statistically significant positive associations between two EUROCAT subgroups.

Anomaly 1	Anomaly 2	Results of review by EUROCAT Coding and Classification Committee	No. of cases with both anomalies	Analyzing multiples only		Analyzing all anomaly cases	
				Odds ratio	Adjusted *p* value	Odds ratio	Adjusted *p* value
Omphalocele	Limb reduction	Limb body wall complex	46	1.59	0.0477	5.35	<0.0001
Encephalocele	Limb reduction	Limb body wall complex	15	2.3	0.0408	2.29	0.012
Ano-rectal atresia/stenosis	Bladder exstrophy	OEIS complex	43	9.53	<0.0001	31.66	<0.0001
Omphalocele	Bladder exstrophy	OEIS complex	43	17.68	<0.001	38.14	<0.001
Spina bifida	Bladder exstrophy	OEIS complex	24	11.77	<0.0001	5.04	<0.0001
VSD	Oesophageal atresia	VACTERL association	165	1.53	0.0003	2.01	<0.0001
Ano-rectal atresia/stenosis	Limb reduction	VACTERL association	79	1.63	0.0028	8.14	<0.0001
Ano-rectal atresia/stenosis	Renal dysplasia	VACTERL association	66	2.81	<0.0001	6.65	<0.0001
Ano-rectal atresia/stenosis	Bilateral renal agenesis	VACTERL association	42	5.03	<0.0001	15.55	<0.0001
Tetralogy of Fallot	Oesophageal atresia	VACTERL association	40	2.49	<0.0001	5.77	<0.0001
PDA as only CHD in term infant	Diaphragmatic hernia	Associated anomalies	22	2.3	0.0095	1.78	0.033
Arhinencephaly/holoprosencephaly	Anophthalmos/microhpthalmos	Associated anomalies	15	8.42	<0.0001	25	<0.0001
Neural tube defects	Omphalocele	Known association	111	5.65	<0.0001	5.23	<0.0001
Anencephalus and similar	Omphalocele	Known association	56	8.47	<0.0001	5.96	<0.0001
Spina bifida	Omphalocele	Known association	45	3.97	<0.0001	3.92	<0.0001
Neural tube defects	Bladder exstrophy	Known association	24	5.17	<0.0001	2.4	0.001
Bilateral renal agenesis	Limb reduction defect	Known association	18	2.12	0.0408	1.6	0.001
Posterior urethral valve	Clubfoot	Sequence	15	2.44	0.0325	0.65	0.195
Ano-rectal atresia/stenosis	Posterior urethral valve	Mainly cases with Prune Belly Sequence (11/14)	14	2.33	0.0497	3.99	<0.0001
Hydrocephalus	Anophthalmos/micropthalmos	Explained by coding errors	21	2.45	0.0056	6.57	<0.0001

*VSD* ventricular septal defect, *PDA* patent ductus arteriosus, *CHD* congenital heart defects.

**Table 2:** Conclusions after literature reviews and individual case review by the EUROCAT Coding and Classification Committee for the remaining 11 statistically significant positive associations between two EUROCAT anomaly subgroups (ordered by odds ratios).

Anomaly 1	Anomaly 2	Comments and conclusions after literature and individual case review by EUROACT Coding and Classification Committee	No. of cases with both anomalies	Analyzing multiples only		Analyzing all anomaly cases		
				Odds ratio	Adj *p* value^a^	Odds ratio	Adj *p* value^a^	% with karyotype known^b^
Encephalocele	Anophthalmos/micropthalmos	7 TOPFA; 6 occipital encephaloceles and 4 nasofrontal encephaloceles. Very few cases are reported in the literature: new association	10	6.74	0.0001	13.15	<0.0001	70
Ebstein’s anomaly	Cleft lip	Association reported in the literature for 2 cases with genetic diagnoses. Some of the 5 cases here may have an undiagnosed genetic disorder. In addition, significance is borderline: not a new association.	5	5.12	0.0437	0.61	0.556	75
Microcephaly	Congenital cataract	10 cases were reported with dysmorphic features or suspected genetic anomaly: not a new association	12	4.17	0.0013	3.12	<0.0001	67
Anophthalmos/micropthalmos	Cleft lip	Majority of cases are diagnosed prenatally. 16 cases had associated cerebral anomalies (hydrocephaly, encephalocele or anomalies of corpus callosum). The association has not been found in the literature: new association	27	3.62	<0.0001	4.43	<0.0001	64
Anencephalus	Gastroschisis	See [9] NTD and gastroschisis below [10]: weak evidence in the literature for association	9	3.36	0.0205	0.39	0.005	38
Hydrocephaly	Hypoplastic right heart	All cases were male. One miscoded Hypoplastic left heart. The remaining cases had other anomalies (6 renal/genital). The literature describes a few cases with this association (mainly pulmonary valve stenosis), but with a genetic background [28, 29] Association of hydrocephaly and variable heart and other defects is also seen in VACTERL-H syndrome [30], but genital anomalies are not part of this: new association	10	3.34	0.0178	2.27	0.039	70
AVSD	Duodenal atresia/stenosis	See severe CHD and duodenal atresia/stenosis below: new association	11	2.65	0.0382	4.81	<0.0001	50
Tetralogy of Fallot	Duodenal atresia/stenosis	See severe CHD and duodenal atresia/stenosis below: new association	11	2.53	0.0476	2.89	0.006	22
Encephalocele	Cleft lip	2 incorrect codes. 10 cases TOPFA. Both anterior and posterior encephaloceles were observed. Few cases of the encephalocele and cleft lip were reported in the literature [11, 12]. Authors of case reports of child with holoprosencephaly, orofacial cleft and fronto-nasal encephalocele discuss if it is a new syndrome [13]: weak evidence in the literature for association	14	2.41	0.0412	0.95	1.0	50
Neural tube defects	Gastroschisis	Association with NTD and omphalocele is well-described [9]. Only 1 report in the literature describing cases of NTD and gastroschisis [10]. Due to the potential misclassification of omphalocele and gastroschisis, the diagnosis of gastroschisis was checked and only 1 was incorrect. Only eight cases with NTD and gastroschisis as the only anomalies: weak evidence in the literature for association	18	2.33	0.0182	0.32	<0.0001	50
Severe CHD	Duodenal atresia/stenosis	The association of non-genetic duodenal atresia and Tetralogy of Fallot or AVSD seems new, but 50% missing karyotype information may explain this finding. There is little published literature, but [26] discusses a familial disorder with duodenal atresia and tetralogy of Fallot: new association	41	2.02	0.0043	1.59	0.022	51

*AVSD* atrioventricular septal defect, *CHD* congenital heart defect, *NTD* neural tube defect.

^a^*P* value adjusted from multiple comparisons using the Benjamini–Hochberg procedure to control the false discovery rate (FDR).

^b^Norway excluded.

There were no combinations of three anomaly subgroups that were statistically significantly more likely to occur than any of the combinations of two anomalies.

### Results of the review by the EUROCAT coding and classification committee

The list of the 31 significant associations was reviewed by the EUROCAT Coding and Classification Committee to determine if the pairs identified were potential unrecognized associations or were part of a known association or sequence or had occurred due to other reasons. Nineteen associations were known associations or sequences already described in the literature (Table 1). Associations were thought to be part of limb body wall complex, OEIS complex (omphalocele-exstrophy-imperforate anus-spinal defects), VACTERL association or sequences like Prune Belly Sequence. One association was explained by coding errors of the anomalies included in the association and related to a known association (also Table 1).

#### Potential new associations

Eleven associations were determined “unknown” and were selected for literature reviews and reviews of the individual cases with the association by the Coding and Classification Committee members in collaboration with the local registries (Table 2). For some associations such as atrioventricular septal defect and duodenal atresia the registries checked for the most recent karyotype testing that may have been performed after the case was notified to the registry. Details of these investigations including the proportion of cases with known karyotypes are given in Table 2 ordered according to the odds ratios.

Two anomaly pairs, microcephaly and congenital cataract; Ebstein’s anomaly and cleft lip, were judged not to be a new association because most of these cases were suspected of having an undiagnosed genetic disorder. Three anomaly pairs were judged to have weak evidence in the literature of a known association: anencephalus and gastroschisis; NTDs and gastroschisis; encephalocele and cleft lip [9–13]. Six anomaly pairs were judged to have evidence of a new association and will be investigated in further detail during annual surveillance in EUROCAT: encephalocele and anophthalmos/micropthalmos; cleft lip and anophthalmos/microphthalmos; hydrocephaly and hypoplastic right heart; atrioventricular septal defect and duodenal atresia/stenosis; tetralogy of Fallot and duodenal atresia/stenosis; severe CHD and duodenal atresia/stenosis.

## Discussion

This study examined 1386 different combinations of two anomalies occurring in the same case and identified 31 significant associations of which 20 were known associations. The remaining 11 significant associations were evaluated in detail and six pairs of anomalies were considered to be new associations and not part of any known association or sequence. The EUROCAT surveillance on these six anomaly pairs will be continued as part of the routine surveillance for clusters and trends.

The classification of significant associations as known associations or sequences was based on published literature. Ten anomaly pairs were part of the limb body wall complex [14, 15], OEIS complex [16, 17] or VACTERL association [18, 19]. The association of neural tube defects and omphalocele was documented previously and explains three of the anomaly pairs found in this study [9, 20]. Newborn infants with diaphragmatic hernia often have pulmonary hypertension [21] which keeps the PDA open explaining one anomaly pair. We classified the anomaly pair posterior urethral valves and clubfoot as the oligohydramnios sequence [22]. The remaining three pairs classified in this study as known associations are less well known but are documented in the literature [23–25].

Of the six new associations, three pairs overlap: severe CHD, Tetralogy of Fallot and common AV canal were found to be associated to duodenal atresia. We only found one publication describing a non-genetic association in two siblings [26]. As this association is very well-described for children with Down syndrome, we will follow up on future cases with a special focus on genetic tests performed. However, it is unlikely that Down syndrome will remain undiagnosed in liveborn infants. The new association between encephalocele and an/microphthalmos was rarely found in the literature [27]. The same holds true for an/microphthalmos and cleft lip, which was not found in the literature. The last new pair of anomalies, hydrocephaly and hypoplastic right heart syndrome, only occurred in males and many cases had associated renal/genital anomalies. We found three publications with these two anomalies together of which two described cases with a genetic background [28, 29] and one suggesting this combination of anomalies could be part of VACTERL [30]. Further surveillance will be done for all new associations.

Other studies have used similar approaches to identify new associations. For example, the Co-occurring defect analysis approach recommended by Benjamin et al. [31] and used by Howley et al. [32], is based on a modified observed-to-expected (O/E) ratio of co-occurring birth defects (congenital anomalies) that was originally proposed by Khoury et al. [33]. The method adjusts for the tendency of birth defects to cluster with other major malformations. The data analyzed in our study firstly only compared the occurrence of a pair of anomalies within cases that had at least two anomalies (whereas the studies above included isolated anomalies) and therefore the tendency to cluster did not need to be adjusted for in the first set of analyses. The second analysis compared pairs of anomalies to cases with only one anomaly and as expected the odds ratios were higher. However, when adjusted *p* values were calculated, a similar set of anomalies was statistically significant at *p* < 0.01. A second important difference between the method adopted by Benjamin et al. [31] was that in this analysis we excluded any cases with known chromosome or genetic anomalies. We wanted to identify any new anomaly clusters—we were not interested in identifying known associations.

The strength of this study was that it was based on data from 32 EUROCAT full-member congenital anomaly registries covering 6,599,765 births between 2008 and 2016. EUROCAT has standardized methods of coding and data cleaning which are adopted by all member registries and the data quality is monitored by the use of data quality indicators. The EUROCAT multiple congenital anomaly algorithm identifies all cases with two or more non-genetic major congenital anomalies in different organ systems, where the pattern of anomalies has not been recognized as part of an association or sequence. A limitation of the study was that it was not possible to obtain detailed genetic information on all cases—the researchers were dependent on the data that had been provided to the registry as the individual cases could not be contacted for more information.

In summary, most associations found by the statistical analysis were known associations already described in the literature. However, there were six new associations that need continued investigation and will be followed by the annual EUROCAT surveillance system.

### Supplementary information

Appendix

## Data Availability

The data analyzed during this study belong to the individual EUROCAT congenital anomaly registries. Applications to analyze these data will be considered by the JRC-EUROCAT management committee and if they are considered of high scientific merit, permissions for the sharing of the data will be sought by the JRC from the relevant registries.

## Code availability

The R data analysis programs are available on reasonable request from JM, the corresponding author.
