# Supplementary material for: Surveillance of multiple congenital anomalies; searching for new associations
Source: Eur J Hum Genet. 2023 Dec 5;32(4):407–12. doi: 10.1038/s41431-023-01502-w (PMC10999451; doi:10.1038/s41431-023-01502-w)
Supplement: Supplementary file 1 — Appendix [file 41431_2023_1502_MOESM1_ESM.docx]

Annex

**Table A**

| **Subgroups and diagnoses within sub-group** | **EUROCAT al codes**  **Included in analysis** |
| --- | --- |
| **Nervous system** |  |
| Neural Tube Defects | al3 |
| Anencephalus and similar | al4 |
| Encephalocele | al5 |
| Spina Bifida | al6 |
| Hydrocephalus | al7 |
| Microcephaly | al8 |
| Arhinencephaly/holoprosencephaly | al9 |
| **Eye** |  |
| Anophthalmos/micropthalmos | al11 |
| Congenital cataract | al13 |
| Congenital glaucoma | al14 |
| **Ear, face and neck** |  |
| Anotia | al16 |
| **Congenital heart defects (CHD)** |  |
| Congenital heart defects | al17 |
| Severe CHD | al97 |
| Common arterial truncus | al18 |
| Transposition of great vessels | al19 |
| Single ventricle | al20 |
| Ventricular septal defect | al21 |
| Atrial septal defect | al22 |
| Atrioventricular septal defect | al23 |
| Tetralogy of Fallot | al24 |
| Tricuspid atresia and stenosis | al25 |
| Ebstein's anomaly | al26 |
| Pulmonary valve stenosis | al27 |
| Pulmonary valve atresia | al28 |
| Aortic valve atresia/stenosis | al29 |
| Hypoplastic left heart | al30 |
| Hypoplastic right heart | al31 |
| Coarctation of aorta | al32 |
| Total anomalous pulm venous return | al33 |
| Patent Ductus Arteriosis as only CHD in term infants (GA≥37 wks) | al100 |
| **Respiratory** |  |
| Choanal atresia | al35 |
| Cystic adenomatous malf of lung | al36 |
| **Oro-facialclefts** |  |
| Cleft lip with or without palate | al102 |
| Cleft palate | al103 |
| **Digestive system** |  |
| Oesophageal atresia with or without tracheo-oesoph | al41 |
| Duodenal atresia or stenosis | al42 |
| Atresia or stenosis of other parts of small intest | al43 |
| Ano-rectal atresia and stenosis | al44 |
| Hirschsprung's disease | al45 |
| Atresia of bile ducts | al46 |
| Annular pancreas | al47 |
| Diaphragmatic hernia | al48 |
| **Abdominalwall defects** |  |
| Gastroschisis | al50 |
| Omphalocele | al51 |
| **Urinary** |  |
| Bilateral renal agenesis including Potter syndrome | al53 |
| Renal dysplasia | al54 |
| Congenital hydronephrosis | al55 |
| Bladder exstrophy and/or epispadia | al56 |
| Posterior urethral valve and/or prune belly | al57 |
| **Genital** |  |
| Hypospadias | al59 |
| **Limb** |  |
| Limb reduction | al62 |
| Clubfoot - talipes equinovarus | al66 |
| Hip dislocation and/or dysplasia | al67 |
| Polydactyly | al68 |
| Syndactyly | al69 |
| **Other anomalies/syndromes** |  |
| Craniosynostosis | al75 |
| Congenital constriction bands/amniotic band | al76 |
| Situs inversus | al79 |
| Conjoined twins | al80 |
| Valproate syndrome | al84 |
